# Lessons Learned and Recommendations from a SCOPE Spinal Cord Injury Neurorestorative Clinical Trials Update

**DOI:** 10.1089/neur.2024.0163

**Published:** 2025-03-05

**Authors:** Bethany R. Kondiles, Sabhya Rana, David Weiner, Armin Blesch, James St. John, Cornelia Haag-Molkenteller, Patrick Freund, James Guest, Daniel D. Mikol, Susan Harkema, Randy D. Trumbower, Michael G. Fehlings, Norbert Weidner, Gary S. Hogge, Edelle C. Field-Fote, Marco A.S. Baptista, Armin Curt, Jane Hsieh, Linda Jones

**Affiliations:** ^1^ International Collaboration on Repair Discoveries, Department of Zoology, University of British Columbia, Vancouver, Canada.; ^2^ Department of Physical Therapy, University of Florida, Gainesville, Florida, USA.; ^3^ Independent Consultant, New Orleans, LA, USA.; ^4^ Department of Neurosciences, University of California-San Diego, and VA San Diego Healthcare System, La Jolla, California, USA.; ^5^ Clem Jones Centre for Neurobiology and Stem Cell Research, Griffith Sciences and Griffith Health, Griffith University, Australia.; ^6^ EG 427, Paris, France.; ^7^ Spinal Cord Injury Center, Research, Balgrist University Hospital, Zürich, Switzerland.; ^8^ Neurological Surgery and The Miami Project to Cure Paralysis, University of Miami, Miami, Florida, USA.; ^9^ NervGen Pharma Corp., Vancouver, Canada.; ^10^ Kessler Foundation, East Hanover, New Jersey, USA.; ^11^ Department of Physical Medicine & Rehabilitation, Spaulding Research Institute, Harvard Medical School, Boston, Massachusetts, USA.; ^12^ Division of Neurosurgery and Spine Program, Department of Surgery, University of Toronto, Toronto, Canada.; ^13^ Spinal Cord Injury Center, Heidelberg University Hospital, Heidelberg, Germany.; ^14^ GSH Biomedical Consulting, San Francisco, California, USA.; ^15^ Hulse Spinal Cord Injury Research, Shepherd Center, Atlanta, Georgia, USA.; ^16^ Christopher & Dana Reeve Foundation, Short Hills, New Jersey, USA.; ^17^ Balgrist University Hospital, Zürich, Switzerland.; ^18^ Wings for Life, Salzburg, Austria.; ^19^ Christopher & Dana Reeve Foundation Short Hills, New Jersey, USA; Thomas Jefferson University, Philadelphia, Pennsylvania, USA.

**Keywords:** biologics, cellular therapy, clinical trials, electrical stimulation, neurorestoration, novel trial design, pharmaceutical, spinal cord injury

## Abstract

The Spinal Cord Outcomes Partnership Endeavors presented a clinical trials update (CTU) in collaboration with the International Spinal Research Trust as a precourse to their annual meeting. Selected trials adhered to *a priori* considerations, prioritizing novelty and a focus on neurorestorative approaches. The sessions featured 13 speakers, covering 4 in-preparation, 4 in-progress, and 4 recently completed trials. In addition to in-person attendance, individuals worldwide viewed a live stream of the presentations. Approximately 1600 participants, comprising clinicians, researchers, industry stakeholders, foundations, and individuals with lived experiences, engaged in the CTU through both in-person and virtual channels. Presentations represented a variety of approaches, including drug, biological, and device-based therapeutics. This summary provides high-level summaries of the trials presented and the resulting discussions including lessons learned. Rather than recapitulating published data, the presentations and discussions emphasized the novelty and strengths of each trial, practical aspects of translation, and lessons learned. Throughout the day, several discussion themes surfaced. These included reflections on the suitability of outcome measures and the distinction between statistically or clinically meaningful effects and meaningful changes in quality of life. Additional topics included novel trial designs, selection of inclusion criteria, recognizing the indispensable role of rehabilitation, tailoring approaches to individual needs, the importance of integrating lived experience, and emphasizing the importance of establishing robust pre-clinical data packages before venturing into clinical translation. Importantly, strategic directives are summarized to address these challenges, focusing resources and efforts to steer forthcoming trials effectively.

## Introduction

The Spinal Cord Outcomes Partnership Endeavors (SCOPE) is an academic industry partnership that aims to enhance the development of clinical trials and clinical practice protocols. SCOPE has sponsored workshops at major scientific meetings on outcome measures, clinical trials, and clinical trial design. This conference proceeding summarizes the most recent clinical trials update (CTU) offered as a precourse to the Annual International Spinal Research Trust (ISRT) meeting. CTUs hold significant promise in shaping the landscape of spinal cord injury (SCI) research.

For example, the International Campaign for Cures of Spinal Cord Injury Paralysis panel was initiated by a call to action in 2004.^1^ The resulting guidelines addressed trial design,^2^ statistical power informed by natural recovery,^3^ outcome measures,^4^ inclusion and exclusion criteria, and ethical considerations.^5^ These article (∼2000 citations in September 2024) influenced the design of subsequent clinical trials.^6^ Analyses of ongoing clinical trials offer an opportunity to identify trial design and outcome advances, as well as challenges to address in future trials.^7–9^

In recent years, there has been a surge in clinical trials employing neurorestorative approaches,^10^ defined herein as restoring function through regeneration or plasticity. Per SCITrialsfinder.net, there were 88 neurorestorative trials in 2016 and 273 in 2023. As the last SCOPE-sponsored CTU occurred in 2016 and with several significant neurorestorative clinical trials either in preparation, in progress, or recently completed, SCOPE recognized the critical need for a CTU. The 2023 CTU was held by SCOPE and ISRT in partnership with Wings for Life and the Christopher & Dana Reeve Foundation, with support from the International Online Spinal Cord Injury Research Seminar (I-OSCIRS). This article summarizes the 2023 CTU presentations and discussions, focusing on novel aspects of each trial, key priorities for advancing therapies, and lessons learned. Finally, drawing from presentations and discussions during the CTU, this article provides strategic recommendations for next steps.

## Trial Identification and CTU Format

A SCOPE subcommittee (Table 1) identified trials for inclusion in the CTU (considerations for inclusion in Box 1). Seven in-preparation trials were considered, with four presented (Table 2). Twenty-five in-progress and 26 completed trials identified from clinicaltrials.gov were discussed for inclusion; five in-progress (Table 3) and four recently completed trials (Table 4) were presented.

**Table 1.** SCOPE Clinical Trial Update Planning Committee Members Represent Academic and Industry Experts in Medicine, Rehabilitation, Clinical Research, and Clinical Trials

Committee member name	Affiliation
Edelle Field-Fote, PT, PhD	Shepherd Center
James Guest, MD, PhD	University of Miami
**Jane Hsieh, MS**	**SCOPE co-Chair**, Wings for Life
**Linda Jones, PT, PhD**	**SCOPE co-Chair**, Thomas Jefferson University, Christopher & Dana Reeve Foundation
Catherine Jutzeler, PhD	ETH Zurich
Steven Kirshblum, MD	Kessler Institute for Rehabilitation
Daniel Mikol, MD, PhD	NervGen Pharma
Kim Pfleeger, PhD	Abbvie

SCOPE, Spinal Cord Outcomes Partnership Endeavors.

**Table 2.** In-Preparation Trials

Trial title	Embryonic stem cell-derived spinal cord neural stem cells for the formation of neural relays	Autologous olfactory nerve bridge transplantation and intensive rehabilitation to repair chronic SCI	Ibudilast-mediated neuroprotection with enhanced plasticity from early pregabalin administration in acute cervical SCI	Dose escalation study of EG110A, administered by intradetrusor injections to adults with neurogenic detrusor overactivity-related incontinence following spinal cord injury who regularly perform clean intermittent catheterization
Presented by	A. Blesch, PhD	J. St. John, PhD	P. Freund, MD, PhD	C. Haag-Molkenteller, MD, PhD
ClinicalTrials.gov ID or comparable	NA	ACTRN12624000391572	NA	NCT06596291
Industry-sponsored or investigator-initiated trial (IIT)	IIT	IIT	IIT	EG 427
Years	TBD	TBD	Initiation planned in 2025	Initiated in 2024
Country	USA	Australia	EU	USA
Planned target population	TBD	TBD	Acute cervical tSCI, AIS A–D	Individuals with SCI and neurogenic detrusor overactivity and urinary incontinence
Mode of administration	Implantation into spinal cord lesion site	Implantation into spinal cord	Oral	One-time injection into the bladder wall via cystoscopy
Putative mechanism of action (broad terms)	Spinal tissue regeneration	Spinal tissue regeneration	Inhibit neuroinflammation and promote axonal regeneration	Block sensory input to dorsal root ganglion to interrupt pathological reflex loop
Putative mechanism of action (specific)	Embryonic stem cells differentiated toward neural stem cells to construct neuronal relays between injured axons and distal neurons	Form nerve bridges to guide regeneration in the spinal cord using OEC that self-assemble using cell-to-cell interactions	Modify inflammation via inhibition of PDE4 using ibudilast, increase plasticity and axonal regeneration by blocking α2δ2 receptors using pregabalin	EG110A is a nonreplicative HSV-1 vector with the transgene encoding the light chain of botulinum toxin F to block the release of neurotransmitter in type C neurons of the DRG. This alleviates neurogenic detrusor overactivity and maintains detrusor muscle function
Proposed primary efficacy end-point	TBD	TBD	Reduction of lesion volume by 20% on MRI	Reduction of urinary incontinence episodes
Key pre-clinical evidence provided by presenter	^11–13^	^14–16^	^17–19^	^20,21^

DRG, dorsal root ganglion; HSV, herpes-simplex virus; MRI, magnetic resonance imaging; OEC, olfactory ensheathing cells; SCI, spinal cord injury; tSCI, traumatic SCI.

**Table 3.** In-Progress Trials

Trial title	Safety and efficacy study of IV administration of Elezanumab to assess change in UEMS in adult participants with acute traumatic cervical SCI (ELASCI)	Study to assess the efficacy and safety of MT-3921 (Unasnemab) in subjects with acute traumatic cervical SCI	A randomized, double-blind, placebo-controlled Phase 1b/2a study of NVG-291 in SCI subjects	Task and physiological-specific stimulation for recovery of autonomic function, voluntary movement, and standing using epidural stimulation and training after severe SCI	Breathing low oxygen to enhance spinal stimulation training and functional recovery in persons with chronic SCI: the BO2ST trial
Presented by	J. Guest, MD, PhD	J. Guest, MD, PhD	D.D. Mikol, MD, PhD	S.J. Harkema, PhD	R. Trumbower, PT, PhD
Industry-sponsored or investigator-initiated trial (IIT)	Abbvie	Mitsubishi Tanabe Pharmaceuticals	NervGen Pharma	IIT	IIT
ClinicalTrials.gov ID	NCT04295538	NCT04683848	NCT05965700	NCT03364660	NCT05563103
Years	2020–2026	2021–2024	2023–2026	2017–2023	2022–2026
Status	Active, not recruiting	Active, not recruiting	Recruiting	Recruiting	Recruiting
Country	USA; Australia; Japan; Israel; Canada; Republic of Korea; Spain	USA, Canada, Japan	USA	USA	USA
Phase	IIa	IIa	Ib/IIa	II	II
Design	Double blinded, randomized, parallel assignment	Randomized, double-blind, placebo-controlled	Randomized, double blind, placebo-controlled	Prospective randomized efficacy study, parallel assignment	Randomized, double-blinded, placebo-controlled, block randomized
Total N	54 (estimated)	72 (estimated)	40 (estimated; 20 in each cohort)	36 (estimated)	48
Target population	Acute (w/in 24 h) cervical (C4-C7) tSCI AIS A/B	Subacute (w/in 3 weeks) cervical (C4-C7) tSCI AIS A/B/C	Cervical motor incomplete tSCI w/in 1–10 years (chronic) or 10–49 days* (subacute) of enrollment *modified to 20–90 days following protocol amendment	>2 years post SCI (chronic), nonprogressive SCI	Chronic incomplete tSCI (>12 months post-SCI), C2-L2, AIS C-D
Mode of administration	i.v.	i.v.	Subcutaneous	Epidural Stimulation Implants	AIH delivered via a nonrebreathing facemask, stimulation delivered via transcutaneous electrodes
Duration and dose of administration	1 year, first dose ≤ 24 h of injury and q4h X 13	First dose <=3 weeks, 6 months (dosage not disclosed)	Daily for 12 weeks (undisclosed dosage)	Two periods of 80 sessions each of designated interventions	8 sessions of room air or hypoxic air breathing over 2 weeks, each session consists of 15 episodes of 1.5-min normoxia or hypoxia. Stim/Sham protocol delivered over a 45 min of gait training session
Putative mechanism of action (broad terms)	Promote axonal regeneration	Promote axonal regeneration	Promote plasticity (primary), axonal regeneration, remyelination	Promote spinal plasticity	Promote spinal plasticity
Putative mechanism of action (specific)	Antibodies bind repulsive guidance molecule A (RGMa), disrupting neogenin downstream signaling. This approach presumably improves spinal cord axonal growth following SCI	MT-3921 is a novel humanized IgG1 monoclonal antibody that binds to RGMa	NVG-291 is a synthetic peptide derived from protein tyrosine phosphatase σ (PTPσ) that has been shown to relieve inhibition of neural repair mediated by glial-derived chondroitin sulfate proteoglycans (CSPGs) following SCI.	Improved task-specific voluntary movements with spinal cord epidural stimulation	Transcutaneous stimulation and AIH induce plasticity, strengthening of neural connections, and improvement in the sensitivity of spinal cord circuitry following SCI
Primary efficacy end-point	UEMS	UEMS	ΔMEP amplitude of first dorsal interosseus and tibialis anterior (coprimary)	Recovery of autonomic control of cardiovascular function and voluntary movement	10 mWT
Additional end-points (if relevant)	SCIM; ΔUEMS	SCIM III; GRASSP; % responders	9HPT; GRASSP; pinch dynamometry force; 10MWT; LEMS; UEMS		
Key pre-clinical evidence provided by presenter	^22,23^	^22,24,25^	^26–29^	Bladder and bowel:^30^	^31,32^

9HPT, 9-hole peg test; 10MWT, 10-min walk test; AIH, acute intermittent hypoxia; AIS, ASIA Impairment Scale; GRASSP, graded redefined assessment of strength sensibility and prehension; LEMS, lower extremity motor score; SCI, spinal cord injury; SCIM, spinal cord independence measure; UEMS, upper extremity motor score.

**Table 4.** Recently Completed Clinical Trials

Trial title	A multicenter, randomized, placebo-controlled, double-blinded, trial of efficacy and safety of riluzole in acute SCI (RISCIS)	Antibodies against Nogo-A to enhance plasticity, regeneration, and functional recovery after SCI—a multicenter international randomized double-blind placebo-controlled Phase II clinical PoC Trial	A Phase 1 safety study of GRNOPC1 in patients with neurologically complete, subacute, SCI: a Phase 1/2a dose escalation study of AST-OPC1 in subjects with subacute cervical SCI	Clinical assessment of upper extremity performance in individuals with SCI using the LIFT system to deliver noninvasive electrical spinal cord stimulation (ARC^EX^ therapy)
Presented by	M. Fehlings, MD, PhD	N. Weidner, MD	G. Hogge, DVM, MS, PhD	E. Field-Fote, PT, PhD
Industry-sponsored or investigator-initiated trial (IIT)	IIT	IIT	Lineage Cell Therapeutics, Inc.	ONWARD Medical Inc.
ClinicalTrials.gov ID	NCT01597518	NCT03935321	NCT01217008; NCT02302157	NCT04697472
Years	2013–2020	2019–2023	2010–2013, 2015–2018	2021–2022
Status	Terminated	Completed	Completed	Completed
Country	Canada, USA, Australia	Czechia, Germany, Spain, Switzerland	USA	USA, Canada, Netherlands, UK
Phase	II and III	II	I; I/IIa	Pivotal
Design	Multicenter, randomized, placebo-controlled, double blinded	Multicenter, randomized, double-blind, placebo-controlled	Both were open label, single-arm trial	Prospective, single-arm open label, sequential assignment
Total N	193 actual (351 planned)	126	5; 25	65 enrolled
Target population	AIS A-C, acute cervical (C4-C8) tSCI, and <12 h post-SCI	Subacute (4–28 days post-SCI), tSCI (C1-T1), AIS A–D	tSCI, AIS A, T3-T11, ZPP <5, ≤14 days post-SCI; tSCI, AIS A, B, C4 to C7, 21–42 days post-SCI	Nonprogressive cervical SCI (C2-C8), AIS B-D, >12 months post-SCI (chronic)
Mode of administration	Oral	Intrathecal	Both via intraparenchymal injection	Transcutaneous spinal cord stimulation over the cervical spinal cord utilizing the LIFT system
Duration and dose of administration	100 mg BID ≤ 24 hours of injury; 50 mg BID for days 2–14 postinjury	6 intrathecal bolus injections, 45 mg of antibody or placebo at each 3.5 mL application	Single injection of oligodendrocyte progenitor cells: 2 million; 2, 10, or 20 million	2 months of 12–20, 1–2 h sessions/month. Total stimulation time ∼60 min during ARC^EX^ therapy
Putative mechanism of action (broad terms)	Prevent secondary damage/promote neuroprotection	Promote axonal regeneration	Both: Remyelination, neurite outgrowth, neurovascularization	Modulation of neural circuitry to facilitate motor output
Putative mechanism of action (specific)	Decrease excitotoxicity and attenuate neurodegeneration following SCI by blocking sodium channels	Antibody that neutralizes Nogo-A, thereby promoting axonal regrowth following SCI	Both: Oligodendrocyte progenitor cells result in myelination, neurite outgrowth, neurovascularization, and fill in cavitation following SCI	Transcutaneous SC stimulation activates afferent SC inputs, altering neural excitability and strengthening existing spinal circuits activated in a task-specific manner to improve function
Primary efficacy end-point	Total UEMS+LEMS; ΔUEMS at 180 days	ΔUEMS	LEMS and sensory scores; UEMS and motor level	Number of device-related SAEs. Composite responder analysis: >50% participants with clinically meaningful improvement in ≥1 of four strength metrics (ISNCSCI, GRASSP strength, pinch force, and grasp force) and ≥1 of two function metrics (CUE-T and GRASSP prehension performance)
Additional end-points (if relevant)	LEMS; AIS; SCIM; ΔNLI; EQ5D index; SF 36; GRASSP	ISNCSCI, autonomic function, SCIM-III, GRASSP, 10MWT, 6MWT, NCV, SSEP NG-101 PK, safety	Both: ISNCSCI	Superiority of combined 2 months FTP (functional task practice training) + 2 months ARC^EX^ therapy +FTP vs. 2 months FTP alone, and preidentified, hierarchically ordered quantitative outcomes. All AEs and SAEs. Effects of ARC^EX^ therapy on SCI sequalae (spasticity, pain, sleep, etc.) and qol metrics
Key pre-clinical provided by presenter	^33–35^	^36–38^	^39–41^	No known animal studies with transcutaneous SCS. First in human tSCS:^42–44^

AE, adverse event; AIS, ASIA Impairment Scale; BID, bis in die (twice a day); CUE-T, capabilities of upper extremity test; EQ5D, EuroQol 5-dimension; GRASSP, graded redefined assessment of strength sensibility and prehension; LEMS, lower extremity motor score; NCV, nerve conduction velocity; NLI, neurological level of injury; qol, quality of life; SAE, serious adverse event; SSEP, somatosensory-evoked potential; SCIM, spinal cord independence measure; UEMS, upper extremity motor score.

Box 1: Considerations for Presented Trials **Considerations for inclusion of all trials presented in the clinical trial update**

Focus on neurorestoration (defined as regeneration or plasticity)

Contains an element of novelty in the trial design, analysis, inclusion criteria, or outcome measures as determined by the planning committee

Include trials across drug, cell, and device-based studies

Trial representative able to present

**Additional considerations for in-progress and completed trials**

SCI trials listed in Clinical Trials.gov after 2016 (last SCOPE clinical trials update), open for screening prior to September 2023 (in-progress trials), and with results prior to September 2023 (completed trials)

Analytics from SCITrialsFinder.net*

**Additional considerations for in-preparation trials**

Likely timing of trial initiation (near term preferred)

*SCITrialsFinder.net produces analytics based on the number of clicks on a given trial and direct contact with a clinical trial via the website. Data on user groups differentiating people with SCI, caregivers, researchers, and clinicians are not collected.

To increase the reach of the CTU beyond the 90 in-person attendees, presentations were live-streamed by the I-OSCIRS. Discussions were held offline to facilitate fluid in-person participation.

Presenters were instructed to focus on lessons learned and novelty as opposed to presenting published results. Neither the presentations nor this summary is intended to serve as an exhaustive scientific review of the background and rationale for each trial and, in several areas, reflect the expert opinion of presenters and participants. Preliminary unpublished results reported during the presentations are noted as such. This article reflects trial status at the time of the CTU, and key updates as of November 2024 are provided. For in-preparation and in-progress clinical trials, refer to trial registration (Tables 2–4) for status and relevant publicly available data.

## Summary of the CTU

### Session 1: A novel biotech perspective

Moderator: Marco Baptista, PhD

Speaker: David Weiner, MD

Dr. Weiner provided a novel perspective on therapeutic drug development for SCI as a consultant working outside SCI. Dr. Weiner, a neurologist trained in neuropharmacology working outside of SCI, has spent 20 years developing innovative therapeutics for multiple neurological disorders and thus provided a novel perspective. He reviewed the well-recognized and urgent need for therapeutics to improve function and address the high burden of care and disability.

Dr. Weiner emphasized that elucidating the underlying biology and pathophysiology is critical to identify relevant targets for therapeutic interventions. Because SCI impacts multiple pathways and cell types, it creates a myriad of opportunities for target identification. However, pathophysiological pathways differ across species,^45^ complicating interpretation of results. Researchers must provide a scientific rationale for relevant targets and include evidence that a given intervention engages and modifies the target or biological pathway of interest. As such, target validation, target engagement, pharmacokinetics (PK), and pharmacodynamics (PD) must be assessed at all stages of the development pipeline. To exemplify this process, Dr. Weiner described the development of the neurite outgrowth inhibitor (Nogo-A) pathway as a target for SCI (Table 5).

**Table 5.** Key Strategies That Should be Employed in SCI Trials to Improve Chances of Identifying Treatments that Promote Recovery

Key strategy	Example in SCI
Target validation Relevance: Does the target play a fundamental role in disease pathophysiology? Activity: Does modulation of the target result in biological, pharmacological, or chemical processes of interest?	Anti-Nogo antibody^46^ -Identified phenotypically - Biology (ligands/receptors) elucidated - Modulation of specific receptor domains stimulated axonal growth - High affinity monoclonal antibodies/peptide antagonists generated and elicit functional recovery *in vivo* - Multiple therapeutic approaches including decoys and Rho inhibitors
Evaluate evidence of target engagement^47^ Characterization of the interaction between a therapeutic agent and its target Dependent on nature of the agent and the biology of the target Defined in cellular systems, *ex vivo*, and *in vivo* in animals (nonclinical) and ideally in humans (difficult); balance difficulty and risk, as assessing in humans counter to derisking Radiolabeling of therapeutic of interest	Anti-Nogo antibody^48^ - IT administration of two Nogo-A antibodies (11C7 and 7B12) at 15 μG/h for 7 days to mice - High level of 7B12 observed at lesion site detected by immunofluorescence - Tissue retention and intracellular translocation observed - Intrathecal administration of a humanized antibody in nonlesioned Macaque spinal cord (C6 level for 8 days)
Pharmacokinetics (PK) Determination of absorption, distribution, metabolism, and excretion of a drug Key metric is exposure, ideally at the site of action, over time Route of administration for SCI needs to address barrier challenges	Anti-Nogo antibody (Humans)^49^ -Phase 1 clinical trial of ATI355, recombinant humanized IgG4 antibody against Nogo-A - Assessed feasibility, safety, tolerability, and PK of IT administration in patients with acute traumatic SCI (ASIA-A) - - Cohorts 1–4—continuous IT infusion (5–30 mg/day), Cohorts 5/6—bolus IT administration (22.5–45 mg) - Exploratory efficacy with neurophysiology (SSEP/MEP/NCV)
Pharmacodynamics (PD) Determination of the physiological, biochemical, or biological effects of a drug Outcomes of interest can vary and are tailored to the drug and the physiology of the disease under study Monitoring exposure is critical Should be assessed at all stages of development from cellular to humans	Riluzole (humans) (not Nogo-A; used as illustrative example)^50^ -Administration of riluzole was associated with a reduction in phosphorylated neurofilament heavy chain (pNF-H), a biomarker that represents axonal degradation.-PK/PD models revealed a positive correlation between riluzole concentration and neurological recovery after 6 months. AXER-204 (Humans)^51^ -Time-dependent and dose-dependent CSF protein changes were observed after Axer-204 administration - Decrease in CSF concentrations, within 1 day, of proteins associated with synaptic organization and axon development, including multiple synaptic adhesion proteins. Anti-Nogo-A antibody (planned analysis) CSF marker proteins to measure the response to Nogo-A (as outcome predictors) will be performed using mass spectrometry-based quantitative proteomic analysis

ASIA, American Spinal Injury Assessment Impairment Scale; CSF, cerebral spinal fluid; IT, intrathecal; MEP, motor-evoked potential; NCV, nerve conduction velocity; SCI, spinal cord injury; SSEP, somatosensory-evoked potential.

Dr. Weiner commented on strategies for establishing therapeutic efficacy. While the translational validity for rodent models of neurological disease is debated, they are useful for defining PK/PD relationships to inform key early clinical trial designs and determine initial target dose(s). Despite a high scientific and regulatory bar for biomarkers as a measure of early efficacy or drug approval, biomarkers offer the potential to correlate initial injury severity with long-term outcomes and enhance prediction models of spontaneous recovery.

Reflecting on future strategies, Dr. Weiner suggested that enhanced predictive capabilities, coupled with refined participant identification methodologies, may improve participant selection. Enrolling likely responders (based on pre-clinical data) in early-stage trials may demonstrate the biological and physiological activity of an intervention. In later-stage trials, prediction and identification models can improve the stratification of larger populations. Ideally, novel clinical outcome measures will be developed, and an immediate goal might be improving existing measures to increase sensitivity and working with regulators on outcomes in registrational trials. A potential opportunity in SCI is regulatory designations to expedite regenerative therapy development and review. For example, Fast Track, Breakthrough Therapy or Device Designation,^52^ or Regenerative Therapy Advanced Designation^53^ are mechanisms designed to accelerate the availability of therapeutic options. Orphan designation^54^ is also an option for approaches with small defined subpopulations (e.g., subacute cervical SCI in ASIA Impairment Scale [AIS] A-C).^1^ Future strategies may become clearer given multiple Phase II studies (some of which target the same pathways) with pending clinical results. Developing technologies, such as viral vector-based approaches and extracellular vehicles, may be options for intervention delivery, pending further study.

### Session 2: In-preparation clinical trials

Moderator: Armin Curt, MD

Speakers: Armin Blesch, PhD; James St. John, PhD; Cornelia Haag-Molkenteller, MD, PhD; Patrick Freund, MD, PhD

Additional details for all trials in this session are found in Table 2.

#### I) Embryonic stem cell-derived spinal cord neural stem cells for the formation of neuronal relays—Armin Blesch, UCSD

A large body of pre-clinical literature provides evidence for the generation of neuronal relays by the implantation of spinal neural stem cells (examples^55,56^). Dr. Blesch focused on scaling up robust pre-clinical strategies (Tuszynski Lab at the University of California at San Diego) into human studies. He described the induction of H9 embryonic stem cells to transition to neuromesodermal progenitor cells, then to neural stem cells, and then dorsal or ventral phenotyping to mimic developmental and anatomical niches.

Dr. Blesch described advances in manufacturing/scale-up practices, safety (including the absence of undifferentiated cells), and cell stability during storage/transport to prepare for clinical use. He showed data describing low variability in cell types produced, consistent genetic markers for appropriate niches, and potency in *in vitro* and *in vivo* assays.^57^ This work is currently in the pre-investigational new drug (IND) stage. Dr. Blesch commented on challenges of obtaining funding necessary for IND enabling and early-stage investigator-initiated trials.

##### Novelty

- Anatomical niche-focused cell differentiation.
- Treatment consists of multicomponent trophic and survival factors combined with cells.

#### II) Autologous olfactory ensheathing cell nerve bridge transplantation and intensive rehabilitation for repairing spinal cord injury—James St. John, Griffith University

Dr. St. John described preparation for a clinical trial to assess autologous olfactory ensheathing cells as bioengineered nerve bridges in the SCI lesion. He described improvements in cell identification, viability, and purity^14^ and individually customized nerve bridges amenable to implantation without the use of scaffolds, matrices, or gels. Dr. St. John showed unpublished data suggesting these bridges integrate with host tissue, retain their migratory and phagocytic capacity, and consistently display appropriate markers after engineering and implantation. Combining implantation with long-term intensive rehabilitation is theorized to guide axonal growth across the lesion.

Unpublished pre-clinical experiments transplanting bridges into mouse transection models show that 1 week after injury implanted animals show motor and sensory improvement, upregulation of genes associated with neural repair pathways, and downregulation of injury pathways. Preliminary histology reflects the ensheathing of axons by implanted cells and axon guidance across the lesion.

##### Novelty

- Three-dimensional (3D) bioengineering of autologous cells.
- Combining implanted bridges and rehabilitation.

#### III) HSV-1 vector gene therapy for neurogenic bladder—Cornelia Haag-Molkenteller, EG 427

Dr. Haag-Molkenteller described EG 427’s plan for a nonreplicative herpes-simplex virus 1 (nrHSV1) gene therapy vector to target sensory neurons of the bladder. Benefits of nrHSV1 over other vectors include targeted approach, large carrying capacity, absence of neutralizing antibody reactions, repeated dosing, and efficient/reliable large-scale manufacturing.

EG 427 is studying two nonreplicative HSV-1 vectors: EG110A to target bladder dysfunction and EG132A to target spasticity. Following SCI, overstimulation of the afferent type-C fibers can cause neurogenic detrusor overactivity (NDO). NDO results in involuntary bladder contractions and urine leakage, leading to increased bladder pressures, urinary tract infections, and decreased quality of life.^58^ After injection into the bladder wall in pre-clinical studies, EG110A travels to the dorsal root ganglia and transduces type C sensory neurons to reduce neurotransmitter release by expression of the light chain of botulinum toxin F.^20^ The current treatment for NDO involves direct BOTOX injection into the bladder muscle, requiring repeat injections every 6–9 months.^58^ EG110A is projected to lead to multiyear efficacy (3 or more) and specificity to sensory neurons/lack of effect on motor neurons. The EG110A intervention holds promise to reduce urinary incontinence, bladder pressures, urinary tract infections, and urinary retention. EG132A is expected to convert excitatory sensory neurons into inhibitory sensory neurons to target spasticity.

A Phase Ib/IIa trial is planned in the United States and United Kingdom* for 2024 to assess safety/efficacy of EG110A. Importantly, EG 427 will include input from individuals with SCI in the trial design.

##### Novelty

- Intervention specifically targets bladder sensory neurons and bladder dysfunction.
- Pinpoint DNA medicine.

##### *Update

- The trial is currently enrolling in the United States (Table 2).

#### IV) Ibudilast-mediated neuroprotection with enhanced plasticity from early pregabalin administration in acute cervical spinal cord injury—Patrick Freund, Balgrist Hospital

Dr. Freund proposed the repurposing of the drug ibudilast to target chronic inflammation and neurotoxicity, with a primary outcome of reducing lesion volume by 20%* and improving upper extremity motor score (UEMS) by >5 points.

Rodent studies utilizing drugs similar to ibudilast show axonal regeneration after SCI.^17^ Human studies in multiple sclerosis demonstrate reduced lesion sizes and increased tissue sparing.^59^ Investigators hypothesize that ibudilast mediates neuroprotection via phosphodiesterase 4 (PDE4) inhibition and reduced inflammation. Gabapentin is prescribed for neuropathic pain, and evidence in humans suggests early administration improves motor recovery,^60^ potentially via enhanced axonal regeneration by blocking α2δ2,^61^ as seen in rodents.

There are no pre-clinical data on ibudilast use in SCI or its combination with gabapentin. But, as both drugs are approved for use in other diseases, this group plans to examine their combined use in SCI. They propose a randomized (1:1) double-blinded Phase IIb trial examining placebo versus ibudilast conducted in the European Multicenter Study about Spinal Cord Injury network to investigate the safety, tolerability, and efficacy of repeated oral doses of Ibudilast (MN-166) in acute traumatic cervical SCI AIS (A–D)*. In addition, they aim to explore additional effects of gabapentinoids as a “standard of care drug” for neuropathic pain (1) within the trial as a secondary objective and (2) in a large associated observational study.

##### Novelty

- Combinatorial repurposed drugs with different proposed mechanisms to modify inflammation.
- Magnetic resonance imaging (MRI)-based measure (lesion width) to assess atrophic changes as primary outcome*.

##### * Update:

- Trial will be a feasibility study assessing UEMS as primary outcome.

#### Challenges and solutions identified during session (i.e., lessons learned)

Several groups described the rigor required to effectively scale up from pre-clinical to clinical deployment, particularly for cell-based interventions. This scale-up is difficult for academic labs and requires significant resources.

Input from individuals with SCI has the potential to improve trial design, enrollment, retention, and participant experience. Early engagement of people with SCI should be included in early trial design to reduce participant burden and focus on outcomes important to people living with SCI.

Utilizing retrospective observational datasets for human data may identify therapeutic approaches that can be evaluated in animal models. Particularly regarding combinatorial approaches, discussion focused on the necessity of a comprehensive understanding of the rationale behind combining treatments and determining the necessary scope and nature of pre-clinical testing for drugs already approved for human use. Historically, human trials have been informed by pre-clinical data in SCI models. Repurposing interventions in the absence of an SCI pre-clinical package may represent a faster path to evaluate potential therapeutics. However, this approach also means trials are initiated with more unknowns, such as mechanisms or interactions, which must be carefully considered. Discussion also focused on strategies to discern the effects of each intervention in trials where randomization of both interventions is not feasible.

### Session 3: In-progress clinical trials

Moderator: Linda Jones, PT, PhD

Speakers: James Guest, MD, PhD; Daniel Mikol, MD, PhD; Susan Harkema, PhD; Randy Trumbower, PT, PhD

Additional details for all trials in this session are found in Table 3.

#### I) Safety and efficacy study of intravenous (IV) administration of Elezanumab to assess change in Upper Extremity Motor Score (UEMS) in adult participants with acute traumatic cervical SCI (ELASCI), Abbvie

#### II) Study to assess the efficacy and safety of MT-3921 in subjects with acute traumatic cervical SCI, Mitsubishi James Guest, University of Miami

Two trials are examining intravenously delivered monoclonal antibodies designed against repulsive guidance molecule A (RGMa) targets. A primary effect of the antibodies is to disrupt RGMa binding to the neogenin receptor. RGMa signaling is upregulated after SCI and inhibits axonal growth via multiple mechanisms.^22^ Further, it affects cell survival and immune responses, making it a potential multimechanistic target.^62^ Importantly, RGMa binding of neogenin activates RhoA kinase signaling and subsequently causes growth cone collapse. Thus, interrupting RGMa binding/neogenin signaling is hypothesized to support axonal plasticity and regeneration.^23^

The Mitsubishi-sponsored study entails IV injections of antibodies (Unasnemab; MT-3921) within 3 weeks of a traumatic cervical SCI and continuing monthly until day 150 (≈ 6 doses). The Abbvie ELASCI study administers antibodies (Elezanumab; AT-555) within 24 h of traumatic cervical SCI and then every 4 weeks, totaling 13 doses. The Mitsubishi study is of shorter duration and includes individuals with AIS A, B, and C, whereas the Abbvie study enrolls AIS A and B. These differences in trial design will be important considerations when results are available. A subset of sites in the ELASCI trial added serial electrophysiological assessments to motor and functional assessments. Longitudinal plasticity of motor circuits will be assessed by motor-evoked potentials (MEPs) and voluntary electromyography.

Dr. Guest highlighted the unique opportunities created by assessing efficacy in different populations with different inclusion criteria. The resulting analyses will be independent and can serve as reciprocal validation. Furthermore, the antibody in each trial targets different epitopes, permitting assessment of the robustness of the target.

### Novelty

- Independent trials validate a single therapeutic approach.
- Long duration of antibody administration, lasting up to a year with Elezanumab.
- Electrophysiology assessments may indicate efficacy by detecting changing signals below clinically measurable motor scores.

Update: both trials active, not recruiting.

#### III) A Phase Ib/IIa study of NVG-291 in individuals with subacute or chronic SCI—Daniel D. Mikol, NervGen

Protein tyrosine kinase phosphatase sigma (PTPσ) is a receptor of chondroitin sulfate proteoglycans that mediate the inhibition of neural repair.^26^ Pre-clinical acute and chronic intervention models of SCI in rodents using intracellular sigma peptide (ISP), a peptide derived from intracellular sequence of rodent PTPσ, demonstrate improved functional (motor, bladder) recovery, with evidence of enhanced plasticity, axonal regeneration, and remyelination.^26–28,63^ Given that most animal models of SCI are motor incomplete, in this proof-of-concept clinical trial, NVG-291, the human analog of ISP, is being tested in participants with subacute and chronic motor incomplete cervical SCI. Although pre-clinical data showed efficacy in both cervical and thoracic injury models, chronic pre-clinical studies were only conducted in cervical injury to date; thus, the clinical trial includes only participants with cervical SCI.

This is a single-center trial designed to maximize the reliability of electrophysiological assessments (the primary outcome) and the consistency of rehabilitation administered to all participants. Inclusion/exclusion criteria are based on minimal and maximal motor function and MEPs, which must be present in two specific qualifying muscles. The fixed dose selected was determined from pre-clinical and healthy subject dosing studies, with exposures in this trial predicted to overlap with or exceed the broad range of efficacious doses from pre-clinical studies. The anticipated outcome of improved neuronal connectivity is assessed by change in overall motor function (hand function and mobility) and MEPs, a surrogate measure of motor recovery. PK and PD (blood proteomics and transcriptomics) data will be collected.

##### Novelty

- Subacute and chronic SCI.
- Electrophysiological assessments (objective, quantitative, continuous, and maximize probability of detecting an efficacy signal) are used to identify potential participants and as outcome measures.

#### IV) Task-specific epidural stimulation study (TS EPI)—Susan Harkema, formerly University of Louisville, now Kessler Foundation

Studies assessing lumbosacral stimulation for locomotor function after SCI identified multiple off-target effects for therapeutic investigation.^64,65^ This study utilizes epidural stimulation at subthreshold levels, previously used to enable locomotion. While the primary outcome is cardiovascular function, a secondary hypothesis aims to determine the importance of stimulation parameter specificity for the intended outcome and combinatorial effects of stimulation with rehabilitation.

Dr. Harkema described protocols for implantation and infection reduction. Pre-implant 3D spine and spinal root mapping aids initial implantation at the targeted spinal segment, and pre-surgical imaging and intraoperative neurophysiology offer fine-tuned placement during surgery. Postoperative spatiotemporal mapping identifies appropriate parameters for targeted motor behavior and physiological responses.

Dr. Harkema reported that thus far, all implanted participants (*n* = 51 across all epidural stimulation trials, *n* = 32 in this trial) can initiate voluntary (not necessarily functional) movement with customized mapping and stimulation parameters. Preliminary data from individuals with cervical SCI and lumbar electrodes showed that targeted cardiovascular stimulation can stabilize systolic blood pressure with a target of 110–120 mm Hg, ameliorating both autonomic dysreflexia and hypotension.^66^ Participants qualitatively noted off-target effects of stimulation with a greater impact on quality of life than motor effects. Additional reported effects include feeling “normal”; temperature regulation; reduced fatigue, spasticity, and caregiver needs; and improved cognition and independence. Four notable related adverse events include one hip fracture, two infections leading to explant, and one instance of pain with stimulation.

##### Novelty

- Examining off-target effects as evidence for clinical indication.

#### V) The BO_2_ST trial: breathing low oxygen to enhance spinal stimulation training and functional recovery in persons with chronic SCI—Randy Trumbower, Harvard Medical School

Both daily acute intermittent hypoxia (AIH) and transcutaneous spinal cord stimulation (tSTIM) have been evaluated in clinical trials^67–69^ to induce neural plasticity. The group hypothesizes that combining these interventions will amplify plasticity and enhance the training-related gains of each. The BO_2_ST trial plans to enroll 64 persons with chronic, motor incomplete SCI to investigate the efficacy (AIH resulting in enduring effects on WALK_tSTIM_), potency (AIH accelerating the benefits of WALK_tSTIM_), and safety of this combined approach on overground walking recovery. The trial aims to determine if the combined interventions will amplify or detract from one another and how they should be delivered temporally (e.g., pretreatment or combination). The study includes the intervention plus intense training, with 1- and 2-month follow-ups.

Preliminary unpublished analyses show that while each intervention’s measured effect is short-lived (days to weeks), the combination allows individuals to reach clinically meaningful effects sooner than with either intervention alone and with more significant, longer-lasting (months to years) gains.

Randomly assigning participants to one of three intervention groups permits adequate controls (comparing combo with either intervention individually). The study can improve recruitment by broad inclusion criteria (as pre-clinical data indicate efficacy across broad ranges), a low burden and commitment for participants, and the promise of 1 month of high-intensity training in all groups. Due in part to the broad inclusion criteria, many covariates complicate decision-making and increase experimental noise (e.g., concurrent medication use, measurement error, placebo effects, etc.). Ongoing work with statisticians aims to determine important covariates in baseline data that might impact outcomes and identify physiological factors (e.g., prevalence of sleep-disordered breathing^70^) common to responders. A second challenge is conducting traditional dosing studies involving combinatorial interventions. As often occurs in stimulation studies, it is hard to control for participants in the stimulation groups who may be aware of the stimulation condition. Finally, the physiological targets underlying the clinical assessments in humans remain unclear.

##### Novelty

- Combining approaches that target complementary mechanisms of neural plasticity may result in greater beneficial effects than either alone.
- Consideration of unintended deleterious interactions.

#### Challenges and solutions identified during session (i.e., lessons learned)

Combinatorial interventions, or the inclusion of intense therapy, creates unique opportunities (such as amplifying task-specific gains) and difficulties (balancing dosing and timing of administration among multiple interventions and differentiating the effects of rehabilitation from the effects of the experimental intervention).

Broad recruitment ranges (e.g., 1–10 years postinjury for chronic SCI in the NVG-291 trial or broad inclusion criteria in the BO_2_ST trial) assist with enrollment numbers but may require stratified analysis due to the potential for increased noise and/or heterogeneity. One strategy to increase enrollment is to ensure the control arm receives an intervention and thus has a potential benefit (e.g., exercise/training in the NVG-291 trial).

### Session 4: Recently completed clinical trials

Moderator: Jane Hsieh, MSc

Speakers: Michael Fehlings, MD, PhD; Norbert Weidner, MD; Gary S. Hogge, DVM, PhD; Edelle Field-Fote, PT, PhD

Additional details for all trials in this session are found in Table 4.

#### I) Riluzole in spinal cord injury study (RISCIS)—Michael Fehlings, University of Toronto

Riluzole, a sodium-glutamate antagonist, is currently an established neuroprotective medication for amyotrophic lateral sclerosis (ALS). A Phase I/IIa trial was conducted in SCI, and based on these findings, a Phase II/III trial was conducted in cervical SCI.^71^

Oral riluzole administration was initiated within 12 hours of injury, a time window infrequently targeted in trials. The trial was terminated before enrollment was complete, but modified subanalyses provided additional information.

Ultimately, 193 patients were randomized into riluzole or placebo with 83% follow-up at 1 year.^50^ There were significantly more participants with AIS A injury severity enrolled, possibly due to the rapid transport of more severe injuries to trial sites. The primary outcomes (UEMS and total motor score) were assessed 180 days after injury and showed participants taking riluzole had a mean difference of 1.76 on UEMS and 2.86 on total motor score versus control.^50^ Dr. Fehlings also detailed the multiple preplanned secondary analyses, including change in neurological level, independence measures, and mental health scores (Table 4) some of which preliminarily showed positive signal. Dr. Fehlings emphasized the importance of developing and utilizing multiple and alternative methods of analysis to gather as much data as possible from clinical trials.

The rate, relatedness, and severity of adverse events did not differ between riluzole and placebo groups. PK/PD data showed an association between motor recovery and drug levels. Furthermore, riluzole is associated with less elevation in levels of phosphorylated neurofilament heavy chain, a biomarker of axonal degradation, as compared with placebo.^72^

Planned alternative and additional analyses include group-based trajectory modeling, recursive partitioning, combining outcome measures to permit global statistical test approaches, examination of MRI findings, and a meta-analysis of Phase I/IIa and II/III.

##### Novelty

- First deployment of a sodium-glutamate antagonist in SCI.
- Repurposing a drug with high safety and tolerability that can be easily administered by first responders.

#### II) Nogo-inhibition in spinal cord injury—first results of a randomized placebo-controlled phase II clinical trial—Norbert Weidner, Heidelberg University Hospital

This multicenter trial evaluated the efficacy of NG101 (anti-Nogo-A antibody) following a Phase I trial that demonstrated safety and feasibility.^49^ The current trial used unbiased recursive partitioning (URP) to predict UEMS recovery at 6 months based on standardized neurological assessments at baseline, which permitted balanced inclusion into predefined subgroups who would not likely reach a ceiling effect in the course of natural recovery and potentially be responders to the intervention. This inclusive approach identified eligibility in ∼73% of participants (AIS A–D).

Over 3 years, 126 participants consented to and were randomized into the study (78 experimental and 48 control); an intention-to-treat analysis showed a small nonsignificant increase in the UEMS (primary outcome) with the Nogo-A antibody. A subgroup analysis showed improved UEMS and functional recovery (spinal cord independence measure self-care; secondary outcome) in participants with motor incomplete (AIS C and D) injuries (unpublished).

The mechanisms by which the anti-Nogo antibody is hypothesized to promote axon growth may explain these results. Meaningful regeneration requires an anatomically incomplete injury with preserved neural tissue across the injury site for sprouting collaterals to reach viable targets. *A priori* stratification defined by URP reflected preserved tissue bridges and more preserved lower motor pools in responder groups compared with nonresponders.

Given the positive findings in the subgroup analysis in the Phase II study, the group plans further studies. Cerebral spinal fluid (CSF) proteomics (unpublished) suggests engagement of a candidate protein important for tissue remodeling, synaptogenesis, and repair, providing an opportunity for validation and mechanistic testing.

##### Novelty

- Effective use of URP to balance participants between treatment arms and identify potential responders.

#### III) Key learnings from dose escalation studies of OPC1 in subacute spinal cord injury (thoracic and cervical)—Gary Hogge, Lineage Cell Therapeutics

In this approach, pluripotent stem cells are terminally differentiated into oligodendrocyte progenitor cells (OPC1) and implanted into the spinal cord to promote myelination, neurite outgrowth, and neovascularization and to prevent/fill in cavitation. An earlier study (sponsored by Geron Corporation) demonstrated a positive safety profile in thoracic SCI.^73^ A Phase I/IIa study (sponsored by Asterias Biotherapeutics) established safety and feasibility in multiple cervical cohorts with escalating doses of cells.^74^ Long-term follow-up will continue to 15 years for both studies, and no serious adverse events have been reported related to OPC1 at the 10-year follow-up of the first study.

Following these trials, significant advances were made in manufacturing and implantation protocols. Improvements include large-scale production with no animal reagents, a ready-to-inject formulation to reduce dose-preparation errors, and a fully functional cell line of higher purity. A novel surgical delivery device for implantation was designed, created, and tested to improve usability while enhancing participant safety. A proof-of-concept study to establish the safety and utility of the new surgical device in 5–10 participants was submitted to the U.S. Food and Drug Administration (FDA).

Dr. Hogge noted the importance of recruiting sites with potential for high-capacity enrollment, representing different regions with a wide geographic catchment area. Accommodating feedback from participants, the sponsor plans to use digital study materials (vs. paper), provide stipends (in compliance with established recommendations), and use SCI specific quality of life questionnaires.

The time window for implantation (21–42 days postinjury) was based on biological data from pre-clinical studies but presents challenges for enrollment. Further, a time window for chronic SCI needs to be established. The optimal control group for cell transplantation is challenged by the ethics of sham surgeries. Age at injury and cause of injury are considerations for participant stratification, and incorporation of standardized rehabilitation programs is needed to identify gains beyond spontaneous recovery. Individuals with C4 injuries present a unique challenge given the severity of the injury and functional loss.

##### Novelty

- Person-mounted magnetic needle in surgical device that moves with ventilation removes need to pause ventilation during implantation.

#### IV) Clinical assessment of upper extremity performance in individuals with spinal cord injury using the LIFT system to deliver noninvasive electrical spinal cord stimulation—Edelle Field-Fote, Shepherd Center

The recently completed Up-LIFT trial assessed the safety and effectiveness of ARC^EX^ therapy, delivered in conjunction with functional task practice training in individuals with tetraplegia.^75^ In a run-in study design, participants received 2 months of upper extremity (UE) functional task practice training followed by 2 months of ARC^EX^ therapy combined with UE functional task practice training.

Initially, 65 participants were enrolled, and at the end of the 4-month study, a modified intention-to-treat (mITT) analysis was conducted on 60 individuals who completed all study procedures. The mITT population included individuals with AIS B, C, and D grade injury, with a larger proportion of individuals in the AIS C and D subgroups. Injury chronicity ranged from 1 to 34 years. The stimulation intensity utilized throughout the trial varied among participants but was tolerated well.

The primary effectiveness end-point was met, with 72% of participants achieving clinically significant improvements in at least one strength and one function metric.^75^ There were no serious device-related adverse events. The secondary end-points assessed improvements beyond the plateau achieved by rehabilitation alone and suggested further improvements are enhanced with concomitant stimulation. Exploratory analyses revealed a higher responder rate in the AIS C and D subgroups; however, those designated as nonresponders may also receive functional benefits. Dr. Field-Fote described small improvements that did not achieve the threshold for clinically significant improvements but greatly impacted an individual’s quality of life.

##### Novelty

- Choice of end-point—composite responder analysis (clinically significant improvement in at least one strength and one function metric) demonstrating improvement in impairment and function with less likelihood of compensation.
- Single-arm run-in study design permits participants to serve as their own control without a crossover design.

#### Challenges and solutions identified during session (i.e., lessons learned)

Presentations in all sessions commented on the lack of tools to address low enrollment and heterogeneous populations. A focus on secondary measures and tools such as URP to stratify participants illustrates how intelligent data-driven design and analysis can increase the signal-to-noise ratio.

Identifying populations most likely to respond based on pre-clinical data or biomarkers may inform proof-of-concept trial design. Despite difficulties in comparing severities and timelines in pre-clinical studies to human SCI, these studies can inform the severity or injury phase most likely to benefit from the intended intervention. Effective screening will identify participants predicted to be responders for reasons such as less severe injury, specific timepoint postinjury, or key anatomical/morphological biomarker (e.g., preserved tissue substrate). Evaluating efficacy in responders may help determine targets or mechanisms that can be further developed for other populations (e.g., more severe injury or chronic timepoint). A key component of this development is confirming a consistent mechanism of action and target engagement across subpopulations.

## Discussion: Challenges and Opportunities

Several significant and long-awaited trials of neuroprotection, regeneration, and neuroplasticity reported findings at this CTU. The in-preparation and in-progress trials are likely to yield vital data in future CTUs. In-person discussions and attendance at meetings provide a critical opportunity to synthesize current efforts and strategize to direct future efforts. A unique feature of this CTU, the online streaming and large virtual audience, suggests there is added utility in increasing the accessibility of CTUs for those who cannot attend in-person meetings. Here, we summarize the discussion and themes that emerged, reflecting the perspectives and opinions of those present. We further contextualize and provide recommendations for consideration for future trials.

### Pre-clinical studies

Attendees vigorously debated the type and extent of pre-clinical data necessary to initiate a trial, including the need for pre-clinical efficacy data in SCI models. There was debate regarding the best use of animal studies for establishing efficacy. Considering small versus large animal models, the discussion centered less on the model used but instead on using the data to derisk Phase I studies. The group largely agreed that large animals more closely model key anatomical or functional pathways and may be necessary for key pre-clinical data that cannot be acquired in rodents. Ideally, biomarkers that exhibit consistency across different species will be identified. Animal models can establish PK/PD, mechanisms of target engagement, and therapeutic windows to provide starting points for clinical studies.

#### Recommendations to derisk the pre-clinical to clinical pipeline

Integrate evidence of mechanistically based target engagement at all stages of the development process.
- Provide resources for investigators to access biotechnology/drug development ecosystem.
- Concerted funding strategies between multiple funders for late stage pre-clinical and early-stage clinical trials.

#### Interventions

Combinatorial interventions may approach the same target through different mechanisms or engage different complementary targets. Combinatorial approaches present unique challenges in determining the timing and dosing of all interventions. Consequently, multimodal approaches require a strong scientific rationale with clearly elucidated mechanisms for all modalities. Ideally, multiple levels of inquiry (i.e., cellular, animal, and human studies) provide insight into the target and how the intervention modifies the target. While the potential for amplified effects with combinatorial approaches is encouraging, the potential for negative interactions between interventions requires additional care and management.

Ongoing development of improved technologies (e.g., stimulation methods) and pipelines (e.g., drug screens and compound development) will produce more specific interventions toward precise targets and reveal key aspects of engagement. For example, it may be more desirable to modulate a receptor as opposed to completely blocking it.

Repurposing approved drugs enlarges the pool of potential therapeutics. Repurposing may offer a faster path to clinical trials, reduce barriers to initial safety studies, and support regulatory approval for human use in SCI.^76^ Previous work establishing the efficacy of these drugs in other conditions may provide insight and a hypothesis for the mechanism of action that can be validated using SCI animal models. Derisking of studies with previously approved therapeutics with robust evidence may attract interest from investors for SCI clinical trials if new intellectual property potential is present.

Thus far, with small sample sizes and focused inclusion/exclusions criteria, the stimulation trials reported effects in most participants. Importantly, some efficacy is seen across the spectrum of injury severity and chronicity, facilitating mechanistic inquiries in SCI subpopulations. It is important that ongoing and future studies validate mechanisms^77^ and inform future work. A remaining challenge in stimulation trials is the difficulty in creating effective placebo conditions to decrease the risk of false positives.

Several studies include targeted rehabilitation. Evidence from pre-clinical and clinical studies suggests that rehabilitation can influence motor outcomes and may amplify functional recovery when combined with other interventions,^78,79^ although specific mechanisms must still be established.

#### Recommendations

- Identify mechanistically relevant SCI targets for repurposed or combinatorial approaches.
- Frameworks are needed to appraise novel and repurposed drugs at all stages (discovery through translation). The Evaluation Subgroup of the SCI Think Tank is eliciting feedback on an evaluation framework for combinatorial approaches.
- Explore methods to address placebo effect and devise sham interventions.
- Develop protocols that can be harmonized across sites in multicenter trials.
- Explore methods to integrate feasible, cost-effective, task-specific rehabilitation into clinical trials.

#### Trial design

Many presentations highlighted ways to improve recruitment such as providing participant benefits in all arms, where possible, and reducing financial or physical burdens of participation. The difficulty of identifying appropriate controls, such as ethically acceptable controls for surgical interventions, was brought up by several presenters. New control group methods such as historical,^80^ registry-matched, or shared controls may improve trial efficiency. Reliable biomarkers may be detectable long before traditional impairment and functionally based measures; they should be considered when designing trials if/when applicable. The incorporation of biomarkers (e.g., fluid-based biomarkers reviewed in^81^) will facilitate use in some trial designs as an indication of the severity of SCI, recovery, and response to an intervention.

When screening acute or subacute participants, upper and lower bounds are frequently set to prevent ceiling effects and floor effects, excluding participants and potentially causing a loss of valuable data. The growing toolset to address heterogeneous samples was also discussed. Ideally, with these tools, early-stage trials will not require restrictive inclusion criteria. Using *a priori* predictive stratification algorithms while including a heterogeneous sample with broad inclusion criteria in mid-stage trials may provide insight into responsive populations. While heterogeneous populations might introduce noise, they also permit researchers to analyze subpopulations based on known effects (e.g., age-dependent effect of hypoxia [reviewed by^82^]) and provide insight into subpopulations that are more likely to respond. Further, it is important to consider biological and physiological states (e.g., participant estrogen levels) as opposed to stratification based solely on age. However, effective strategies for managing numerous comorbidities, medications, and daily habits (e.g., caffeine intake) have yet to be established. Trial designs that accommodate multiple features, with key input from biostatisticians, are a first step toward identifying confounding factors.

Repeated discussion considered the use of AIS grade as an inclusion criterion. The International Standards for Neurological Classification of SCI (ISNCSCI) exam was developed to promote consistent communication between clinicians and researchers in defining neurological levels and severity of injury and is based on motor and sensory scores, including anal.^83^ It has undergone many revisions with input from both clinicians and researchers.^83^ Aspects of the ISNCSCI are commonly used for trial inclusion, as recovery predictors and trial outcomes, to describe heterogeneity and for stratification. For neuroprotective studies where interventions must be given within hours of injury, one must consider clinically feasible, accurate alternatives.

The use of change in AIS grades as an outcome measure was also discussed. AIS is an ordinal measure (as with most measures in SCI), with large “categories” that are not always sensitive to change nor reflective of changes in function. Discussion also centered around the use of motor scores, which might not capture meaningful clinical benefits. Improvement in motor scores can be more or less sensitive than existing functional assessments but, depending on the distribution and magnitude of change, might not always correlate with a commensurate change in function. As motor scores may not be sensitive enough, secondary measures may detect additional effects. For example, small improvements of voluntary motor control measured with electromyography that correlate with meaningful quality of life improvement may differ from functional motor control as defined by a prehension score. However, commonly used measures may fail to capture real quality of life effects or unintended effects (positive or negative) of an intervention on other systems. Thus, more holistic measures should be considered in addition to primary outcome measures. Researchers might consider leveraging multiple data sources to validate results. The current climate, wherein multiple completed trials can be analyzed in concert, may signal a nexus point to gather thought leaders to rethink how we analyze and interpret clinical trials, building on lessons learned.

#### Recommendations

- Continue to build large, accessible pre-clinical and clinical trial datasets and registries to:
  o characterize spontaneous recovery
  o model adaptive trial designs
  o develop precise predictive algorithms
  o explore historical controls or digital twins^84^
  o federate independent datasets
- Aggregate clinical trial data, as in ALS,^85^ to detect trends and off-target effects that impact recovery, generating novel targets and research questions
- Explore and expand novel trial designs (e.g., adaptive and platform trials^86^)
- Convene a biostatistical study group to develop recommendations for:
  o group-based trajectory modeling
  o URP
  o global statistical test approaches on combined outcome measures
  o meta-analyses of Phase I/IIa data
- Pursue development, validation, and regulatory approval of biomarkers to provide an objective determination of severity in earlier stages and for early readouts to support adaptive trial designs.
  o Consider biomarker-focused CTUs/workshops.

#### Effect sizes and measures

Many studies show small effects using current measures, in part due to heterogeneity of trial participants. A key message was the potential for additive small effects in different body systems to collectively and meaningfully impact quality of life, which might not be detected with functionally based outcomes of clinically meaningful improvements. The need for modification of existing measures or the development of more sensitive measures was discussed. Although linear scales, such as the 10-m and 6-min walk test, are commonly used, many clinical outcome assessments are ordinal with inferred ranking (e.g., task A is more challenging than task B) that cannot ascertain the degree of difference in difficulty between tasks. Despite their established value, ordinal measures are restricted in the type, accuracy, and strength of statistical analyses, and comparing outcomes across differing severities and level of injury is difficult.^87,88^

Electrophysiology may prove a promising proof-of-concept biomarker in early-stage trials, as changes in electrophysiology may be detectable before clinically detectable motor effects. While these findings should not be overinterpreted, the ability to assess signal in a person with incomplete injury is a key strength. Discussion revolved around strategies to address concerns of quantification and high variability when using electrophysiology assessments as outcome measures, such as photographing electrode placement and regular remapping. Importantly, tracking these changes over time might yield effects and future avenues of research and therapeutics that might ultimately correlate with other clinical features (e.g., force generated).

Enrolling individuals with less severe injuries and more residual tissue may result in larger observed effect sizes, determine mechanisms of improvement, and elucidate better targets for individuals with more severe injuries and less spared tissue. The increased number of trials incorporating electrophysiology and imaging provides an opportunity to learn from these trials.

#### Recommendations

- Use advanced methodologies such as item response theory (IRT), including Rasch analysis,^89–92^ to evaluate existing measures to assess their underlying constructs, floor and ceiling effects, and gaps in content. IRT may:
  o develop new measures if warranted.
  o quantify differences in level of difficulty between specific test items and scores in existing measures.
- Consider a study group to:
  o Validate the association of patterns or changes in electrophysiological measures with clinical measures and outcomes.
  o Develop robust protocols to normalize data, reduce noise, and standardize across participants and sites as some research networks have done.

#### Lived experience perspective

Many presenters reported activities to increase meaningfully incorporation of lived experiences in the research pipeline, including input on trial measures. Funding agencies increasingly recognize the critical importance of lived experience perspective on grant applications, and some now incorporate this perspective on grant review panels. Many of the presentations included descriptions of participant-reported increased quality of life despite results that fell short of clinical significance. Incorporating quality of life and participant perspectives as outcomes may be a way to detect previously undetected effects as well as centralize the lived experience perspective.

#### Recommendations

The North American SCI Consortium (NASCIC) SCI research advocacy course creates a pool of trained research partners and advisors; investigators should pursue relationships with these partners.
Implement recommendations from upcoming patient-focused drug development (PFDD) meeting with the FDA to express patient preferences for therapeutic development. Ultimately, this PFDD will help inform the field and the FDA of what is important to people living with SCI. Key priorities have been outlined by NASCIC and SCOPE for consideration, including:
  o small effects (vs. clinical effects) on quality of life
  o population heterogeneity
  o appropriateness of outcomes measures
- Identify measures to capture small, meaningful changes outside the primary outcome.

## Conclusion

Presentations at the CTU described advances in pre-clinical studies, interventions, trial designs, and measurements. With insights gleaned from recently concluded trials and the advancements in assessment and analysis techniques, the present moment offers an opportunity to comprehensively consider the field’s status and opportunities for advancement, as outlined by points raised in this article.

Trends stemming from completed trials, revised for ongoing trials, and carefully reconsidered for in-preparation trials may signal the need to review and adjust the trajectory for future trials. The lifespans of individuals living with SCI have not improved over the decades,^93^ and our research community has not yet achieved substantial, functionally significant improvements in neurorestoration. We have identified new and repurposed potential therapeutics (individually and combined), innovative trial designs, novel methods for data analysis, the need to improve existing outcome measures, and develop more specific and sensitive measures. These are green flags suggesting the need to gather clinicians, researchers, people with lived experience, and organizations to strategize for maximal usage of this emerging knowledge base. As the field begins to assemble to tackle lessons learned, we must prioritize additional ongoing consistent efforts to address the remaining gaps.

## Transparency, Rigor, and Reproducibility Statement

This conference proceeding summarizes presentations and discussions held during the Clinical Trials Update precourse held immediately in advance of the 2023 International Spinal Research Trust yearly meeting. In-person attendance was open to all conference registrants. The online stream was publicly available. Attendees represented people with lived experience, funders, and academic and industry experts in medicine, rehabilitation, clinical research, and clinical trials. The methods for determining presenters are found in the “Approach and Trial Identification” section. Recommendations presented herein have been reviewed by all coauthors and represent the discussions on the day, with additional context from the literature.

## Notes

^1^
A number of trial sponsors presented at the CTU received either expedited regulatory designations and/or orphan designation including Abbvie, Lineage, NervGen, Onward, and Mitsubishi.

## Abbreviations Used

9HPT: 9-hole peg test
10MWT: 10-min walk test
AE: adverse event
AIH: acute intermittent hypoxia
AIS: ASIA Impairment Scale
ALS: amyotrophic lateral sclerosis
ASIA: American Spinal Injury Association Impairment Scale
BID: bis in die (twice a day)
CDMRP SCIRP: Spinal Cord Injury Research Program
CSF: cerebrospinal fluid
CTU: Clinical Trials Update
CUE-T: Capabilities of Upper Extremity Test
DRG: dorsal root ganglion
ELASCI: Elezanumab for Acute Spinal Cord Injury
EQ-5D: EuroQol 5-Dimension Health Index
FDA: U.S. Food and Drug Administration
GRASSP: Graded Redefined Assessment of Strength, Sensibility, and Prehension
HSV: herpes simplex virus
IIT: Investigator-Initiated Trial
IND: Investigational New Drug
IT: intrathecal
LEMS: lower extremity motor score
MEP: motor-evoked potential
MRI: magnetic resonance imaging
NCV: nerve conduction velocity
NLI: neurological level of injury
NogoA: neurite outgrowth inhibitor A
OEC: olfactory ensheathing cells
OPC: oligodendrocyte progenitor cells
PDE4: phosphodiesterase 4
PD: pharmacodynamics
PK: pharmacokinetics
PoC Trial: Proof of Concept Trial
qol: quality of life
RGMa: repulsive guidance molecule A
RISCIS: Riluzole in Spinal Cord Injury Study
SAE: serious adverse event
SCI: spinal cord injury
SCIM: spinal cord independence measure
SCOPE: Spinal Cord Outcomes Partnership Endeavors
SSEP: somatosensory-evoked potential
tSCI: traumatic SCI
tSTIM: transcutaneous spinal cord stimulation
TBD: to be determined
TS EPI: task-specific epidural stimulation
UEMS: upper extremity motor score

## References

[1] Adams M, Cavanagh JFR. International Campaign for Cures of Spinal Cord Injury Paralysis (ICCP): Another step forward for spinal cord injury research. Spinal Cord 2004;42(5):273–280; doi: 10.1038/sj.sc.3101597 14968108

[2] Lammertse D, Tuszynski MH, Steeves JD, et al. International Campaign for Cures of Spinal Cord Injury Paralysis. Guidelines for the conduct of clinical trials for spinal cord injury as developed by the ICCP panel: Clinical trial design. Spinal Cord 2007;45(3):232–242; doi: 10.1038/sj.sc.3102010 17179970 PMC4106695

[3] Fawcett JW, Curt A, Steeves JD, et al. Guidelines for the conduct of clinical trials for spinal cord injury as developed by the ICCP panel: Spontaneous recovery after spinal cord injury and statistical power needed for therapeutic clinical trials. Spinal Cord 2007;45(3):190–205; doi: 10.1038/sj.sc.3102007 17179973

[4] Steeves JD, Lammertse D, Curt A, et al. International Campaign for Cures of Spinal Cord Injury Paralysis. Guidelines for the conduct of clinical trials for spinal cord injury (SCI) as developed by the ICCP panel: Clinical trial outcome measures. Spinal Cord 2007;45(3):206–221; doi: 10.1038/sj.sc.3102008 17179972

[5] Tuszynski MH, Steeves JD, Fawcett JW, et al. International Campaign for Cures of Spinal Cord Injury Paralysis. Guidelines for the conduct of clinical trials for spinal cord injury as developed by the ICCP Panel: Clinical trial inclusion/exclusion criteria and ethics. Spinal Cord 2007;45(3):222–231; doi: 10.1038/sj.sc.3102009 17179971

[6] Sorani MD, Beattie MS, Bresnahan JC. A quantitative analysis of clinical trial designs in spinal cord injury based on ICCP guidelines. J Neurotrauma 2012;29(9):1736–1746; doi: 10.1089/neu.2011.2162 22369673 PMC6463987

[7] Badhiwala JH, Wilson JR, Kwon BK, et al. A review of clinical trials in spinal cord injury including biomarkers. J Neurotrauma 2018;35(16):1906–1917; doi: 10.1089/neu.2018.5935 29888678

[8] Donovan J, Kirshblum S. Clinical trials in traumatic spinal cord injury. Neurotherapeutics 2018;15(3):654–668; doi: 10.1007/s13311-018-0632-5 29736858 PMC6095794

[9] Blight AR, Hsieh J, Curt A, et al. The challenge of recruitment for neurotherapeutic clinical trials in spinal cord injury. Spinal Cord 2019;57(5):348–359; doi: 10.1038/s41393-019-0276-2 30962518

[10] Dietz VA, Roberts N, Knox K, et al. Fighting for recovery on multiple fronts: The past, present, and future of clinical trials for spinal cord injury. Front Cell Neurosci 2022;16:977679; doi: 10.3389/fncel.2022.977679 36212690 PMC9533868

[11] Lu P, Woodruff G, Wang Y, et al. Long-distance axonal growth from human induced pluripotent stem cells after spinal cord injury. Neuron 2014;83(4):789–796; doi: 10.1016/j.neuron.2014.07.014 25123310 PMC4144679

[12] Kumamaru H, Kadoya K, Adler AF, et al. Generation and post-injury integration of human spinal cord neural stem cells. Nat Methods 2018;15(9):723–731; doi: 10.1038/s41592-018-0074-3 30082899

[13] Rosenzweig ES, Brock JH, Lu P, et al. Restorative effects of human neural stem cell grafts on the primate spinal cord. Nat Med 2018;24(4):484–490; doi: 10.1038/nm.4502 29480894 PMC5922761

[14] Reshamwala R, Murtaza M, Chen M, et al. Designing a clinical trial with olfactory ensheathing cell transplantation-based therapy for spinal cord injury: A position paper. Biomedicines 2022;10(12); doi: 10.3390/biomedicines10123153 PMC9776288 36551909

[15] Chen M, Shah MP, Shelper TB, et al. Naked liquid marbles: A robust three-dimensional low-volume cell-culturing system. ACS Appl Mater Interfaces 2019;11(10):9814–9823; doi: 10.1021/acsami.8b22036 30724549

[16] Oieni F, Reshamwala R, St John J. Olfactory ensheathing cells for spinal cord injury: The cellular superpowers for nerve repair. Neuroglia 2022;3(4):139–143.

[17] Iannotti CA, Clark M, Horn KP, et al. A combination immunomodulatory treatment promotes neuroprotection and locomotor recovery after contusion SCI. Exp Neurol 2011;230(1):3–15; doi: 10.1016/j.expneurol.2010.03.010 20338167

[18] Costa LM, Pereira JE, Filipe VM, et al. Rolipram promotes functional recovery after contusive thoracic spinal cord injury in rats. Behav Brain Res 2013;243:66–73; doi: 10.1016/j.bbr.2012.12.056 23295392

[19] Myers SA, Gobejishvili L, Saraswat Ohri S, et al. Following spinal cord injury, PDE4B drives an acute, local inflammatory response and a chronic, systemic response exacerbated by gut dysbiosis and endotoxemia. Neurobiol Dis 2019;124:353–363; doi: 10.1016/j.nbd.2018.12.008 30557659 PMC6445388

[20] Joussain C, Le Coz O, Pichugin A, et al. Botulinum neurotoxin light chains expressed by defective herpes simplex virus type-1 vectors cleave snare proteins and inhibit cgrp release in rat sensory neurons. Toxins (Basel) 2019;11(2); doi: 10.3390/toxins11020123 PMC6409900 30791373

[21] Ratelade J, Faugeroux J, Behr-Roussel D, et al. Intravesical administration of Eg110a, a novel non-replicative Herpes simplex virus type 1 (Hsv1)-derived vector expressing the light chain of botulinum toxin F, inhibits C-type fibers in an acute intravesical capsaicin rat model. Continence 2023;7:100817; doi: 10.1016/j.cont.2023.100817

[22] Schwab JM, Conrad S, Monnier PP, et al. Spinal cord injury-induced lesional expression of the repulsive guidance molecule (RGM). Eur J Neurosci 2005;21(6):1569–1576; doi: 10.1111/j.1460-9568.2005.03962.x 15845084

[23] Jacobson PB, Goody R, Lawrence M, et al. Elezanumab, a human anti-RGMa monoclonal antibody, promotes neuroprotection, neuroplasticity, and neurorecovery following a thoracic hemicompression spinal cord injury in non-human primates. Neurobiol Dis 2021;155:105385; doi: 10.1016/j.nbd.2021.105385 33991647

[24] Mothe AJ, Tassew NG, Shabanzadeh AP, et al. RGMa inhibition with human monoclonal antibodies promotes regeneration, plasticity and repair, and attenuates neuropathic pain after spinal cord injury. Sci Rep 2017;7(1):10529; doi: 10.1038/s41598-017-10987-7 28874746 PMC5585220

[25] Nakagawa H, Ninomiya T, Yamashita T, et al. Treatment with the neutralizing antibody against repulsive guidance molecule-a promotes recovery from impaired manual dexterity in a primate model of spinal cord injury. Cereb Cortex 2019;29(2):561–572; doi: 10.1093/cercor/bhx338 29315368

[26] Lang BT, Cregg JM, DePaul MA, et al. Modulation of the proteoglycan receptor PTPσ promotes recovery after spinal cord injury. Nature 2015;518(7539):404–408; doi: 10.1038/nature13974 25470046 PMC4336236

[27] Milton AJ, Kwok JCF, McClellan J, et al. Recovery of forearm and fine digit function after chronic spinal cord injury by simultaneous blockade of inhibitory matrix chondroitin sulfate proteoglycan production and the receptor PTPsigma. J Neurotrauma 2023;40(23–24):2500–2521; doi: 10.1089/neu.2023.0117 37606910 PMC10698859

[28] Tran AP, Sundar S, Yu M, et al. Modulation of receptor protein tyrosine phosphatase sigma increases chondroitin sulfate proteoglycan degradation through cathepsin B secretion to enhance axon outgrowth. J Neurosci 2018;38(23):5399–5414; doi: 10.1523/JNEUROSCI.3214-17.2018 29760175 PMC5990985

[29] Rink S, Arnold D, Wohler A, et al. Recovery after spinal cord injury by modulation of the proteoglycan receptor PTPsigma. Exp Neurol 2018;309:148–159; doi: 10.1016/j.expneurol.2018.08.003 30118740

[30] Hoey RF, Medina-Aguiñaga D, Khalifa F, et al. Thoracolumbar epidural stimulation effects on bladder and bowel function in uninjured and chronic transected anesthetized rats. Sci Rep 2022;12(1):2137; doi: 10.1038/s41598-022-06011-2 35136100 PMC8826941

[31] Lovett-Barr MR, Satriotomo I, Muir GD, et al. Repetitive intermittent hypoxia induces respiratory and somatic motor recovery after chronic cervical spinal injury. J Neurosci 2012;32(11):3591–3600; doi: 10.1523/JNEUROSCI.2908-11.2012 22423083 PMC3349282

[32] Gad P, Choe J, Shah P, et al. Sub-threshold spinal cord stimulation facilitates spontaneous motor activity in spinal rats. J Neuroeng Rehabil 2013;10:108; doi: 10.1186/1743-0003-10-108 24156340 PMC4016220

[33] Schwartz G, Fehlings MG. Evaluation of the neuroprotective effects of sodium channel blockers after spinal cord injury: Improved behavioral and neuroanatomical recovery with riluzole. J Neurosurg 2001;94(2 Suppl):245–256; doi: 10.3171/spi.2001.94.2.0245 11302627

[34] Schwartz G, Fehlings MG. Secondary injury mechanisms of spinal cord trauma: A novel therapeutic approach for the management of secondary pathophysiology with the sodium channel blocker riluzole. Prog Brain Res 2002;137:177–190; doi: 10.1016/s0079-6123(02)37016-x 12440368

[35] Wu Y, Satkunendrarajah K, Teng Y, et al. Delayed post-injury administration of riluzole is neuroprotective in a preclinical rodent model of cervical spinal cord injury. J Neurotrauma 2013;30(6):441–452; doi: 10.1089/neu.2012.2622 23517137 PMC3696918

[36] Schwab ME. Nogo and axon regeneration. Curr Opin Neurobiol 2004;14(1):118–124; doi: 10.1016/j.conb.2004.01.004 15018947

[37] Schwab ME. Functions of Nogo proteins and their receptors in the nervous system. Nat Rev Neurosci 2010;11(12):799–811; doi: 10.1038/nrn2936 21045861

[38] Schwab ME, Strittmatter SM. Nogo limits neural plasticity and recovery from injury. Curr Opin Neurobiol 2014;27:53–60; doi: 10.1016/j.conb.2014.02.011 24632308 PMC4122629

[39] Zhang YW, Denham J, Thies RS. Oligodendrocyte progenitor cells derived from human embryonic stem cells express neurotrophic factors. Stem Cells Dev 2006;15(6):943–952; doi: 10.1089/scd.2006.15.943 17253955

[40] Priest CA, Manley NC, Denham J, et al. Preclinical safety of human embryonic stem cell-derived oligodendrocyte progenitors supporting clinical trials in spinal cord injury. Regen Med 2015;10(8):939–958; doi: 10.2217/rme.15.57 26345388

[41] Manley NC, Priest CA, Denham J, et al. Human Embryonic Stem Cell-Derived Oligodendrocyte Progenitor Cells: Preclinical Efficacy and Safety in Cervical Spinal Cord Injury. Stem Cells Transl Med 2017;6(10):1917–1929; doi: 10.1002/sctm.17-0065 28834391 PMC6430160

[42] Freyvert Y, Yong NA, Morikawa E, et al. Engaging cervical spinal circuitry with non-invasive spinal stimulation and buspirone to restore hand function in chronic motor complete patients. Sci Rep 2018;8(1):15546; doi: 10.1038/s41598-018-33123-5 30341390 PMC6195617

[43] Gad PN, Kreydin E, Zhong H, et al. Non-invasive neuromodulation of spinal cord restores lower urinary tract function after paralysis. Front Neurosci 2018;12:432; doi: 10.3389/fnins.2018.00432 30008661 PMC6034097

[44] Inanici F, Samejima S, Gad P, et al. Transcutaneous electrical spinal stimulation promotes long-term recovery of upper extremity function in chronic tetraplegia. IEEE Trans Neural Syst Rehabil Eng 2018;26(6):1272–1278; doi: 10.1109/tnsre.2018.2834339 29877852 PMC6986544

[45] Filipp M, Travis B, Henry S, et al. Differences in neuroplasticity after spinal cord injury in varying animal models and humans. Neural Regen Res 2019;14(1):7–19; doi: 10.4103/1673-5374.243694 30531063 PMC6263009

[46] McGee AW, Strittmatter SM. The Nogo-66 receptor: Focusing myelin inhibition of axon regeneration. Trends Neurosci 2003;26(4):193–198; doi: 10.1016/S0166-2236(03)00062-6 12689770

[47] Simon GM, Niphakis MJ, Cravatt BF. Determining target engagement in living systems. Nat Chem Biol 2013;9(4):200–205; doi: 10.1038/nchembio.1211 23508173 PMC4004587

[48] Weinmann O, Schnell L, Ghosh A, et al. Intrathecally infused antibodies against Nogo-A penetrate the CNS and downregulate the endogenous neurite growth inhibitor Nogo-A. Mol Cell Neurosci 2006;32(1–2):161–173; doi: 10.1016/j.mcn.2006.03.007 16697217

[49] Kucher K, Johns D, Maier D, et al. First-in-man intrathecal application of neurite growth-promoting anti-nogo- a antibodies in acute spinal cord injury. Neurorehabil Neural Repair 2018;32(6–7):578–589; doi: 10.1177/1545968318776371 29869587

[50] Fehlings MG, Moghaddamjou A, Harrop JS, et al. Safety and efficacy of riluzole in acute spinal cord injury study (RISCIS): A multi-center, randomized, placebo-controlled, double-blinded trial. J Neurotrauma 2023;40(17–18):1878–1888; doi: 10.1089/neu.2023.0163 37279301 PMC10460693

[51] Maynard G, Kannan R, Liu J, et al. Soluble nogo-receptor-Fc decoy (AXER-204) in patients with chronic cervical spinal cord injury in the USA: A first-in-human and randomised clinical trial. Lancet Neurol 2023;22(8):672–684; doi: 10.1016/S1474-4422(23)00215-6 37479373 PMC10410101

[52] U.S. Food and Drug Administration. Fast Track, Breakthrough Therapy, Accelerated Approval, Priority Review. n.d.

[53] U.S. Food and Drug Administration. Regenerative Medicine Advanced Therapy Designation. n.d. Available from: https://www.fda.gov/vaccines-blood-biologics/cellular-gene-therapy-products/regenerative-medicine-advanced-therapy-designation [Last accessed: December 6, 2024].

[54] U.S. Food and Drug Administration. Designating an Orphan Drug or Biologic. n.d. Available from: https://www.fda.gov/industry/medical-products-rare-diseases-and-conditions/designating-orphan-product-drugs-and-biological-products [Last accessed: May 8, 2024].

[55] Ceto S, Sekiguchi KJ, Takashima Y, et al. Neural stem cell grafts form extensive synaptic networks that integrate with host circuits after spinal cord injury. Cell Stem Cell 2020;27(3):430–440.e5; doi: 10.1016/j.stem.2020.07.007 32758426 PMC7484050

[56] Lu P, Gomes-Leal W, Anil S, et al. Origins of neural progenitor cell-derived axons projecting caudally after spinal cord injury. Stem Cell Reports 2019;13(1):105–114; doi: 10.1016/j.stemcr.2019.05.011 31204300 PMC6626851

[57] Li Y, Kumamaru H, Vokes TJ, et al. An improved method for generating human spinal cord neural stem cells. Exp Neurol 2024;376(April):114779; doi: 10.1016/j.expneurol.2024.114779 38621449

[58] Taweel WA, Seyam R. Neurogenic bladder in spinal cord injury patients. Res Rep Urol 2015;7:85–99; doi: 10.2147/RRU.S29644 26090342 PMC4467746

[59] Fox RJ, Coffey CS, Conwit R, et al. NN102/SPRINT-MS Trial Investigators. Phase 2 trial of ibudilast in progressive multiple sclerosis. N Engl J Med 2018;379(9):846–855; doi: 10.1056/nejmoa1803583 30157388 PMC6172944

[60] Warner FM, Cragg JJ, Jutzeler CR, et al. Association of timing of gabapentinoid use with motor recovery after spinal cord injury. Neurology 2020;95(24):E3412–E3419; doi: 10.1212/WNL.0000000000010950 32989101 PMC7836656

[61] Sun W, Larson MJE, Kiyoshi CM, et al. Gabapentinoid treatment promotes corticospinal plasticity and regeneration following murine spinal cord injury. J Clin Invest 2020;130(1):345–358; doi: 10.1172/JCI130391 31793909 PMC6934190

[62] Fujita Y, Yamashita T. The roles of RGMa-neogenin signaling in inflammation and angiogenesis. Inflamm Regen 2017;37(1):6–7; doi: 10.1186/s41232-017-0037-6 29259705 PMC5725648

[63] Shen Y, Tenney AP, Busch SA, et al. PTPs Is a Receptor for Chondroitin Sulfate Proteoglycan, an Inhibitor of Neural Regeneration. Science 2009;326(5952):592–596. 19833921 10.1126/science.1178310 PMC2811318

[64] Aslan SC, Legg Ditterline BE, Park MC, et al. Epidural spinal cord stimulation of lumbosacral networks modulates arterial blood pressure in individuals with spinal cord injury-induced cardiovascular deficits. Front Physiol 2018;9(MAY):565–511; doi: 10.3389/fphys.2018.00565 29867586 PMC5968099

[65] Harkema SJ, Legg Ditterline B, Wang S, et al. Epidural spinal cord stimulation training and sustained recovery of cardiovascular function in individuals with chronic cervical spinal cord injury. JAMA Neurol 2018;75(12):1569–1571; doi: 10.1001/jamaneurol.2018.2617 30242310 PMC6583212

[66] Harkema SJ, Wang S, Angeli CA, et al. Normalization of blood pressure with spinal cord epidural stimulation after severe spinal cord injury. Front Hum Neurosci 2018;12(March):83–11; doi: 10.3389/fnhum.2018.00083 29568266 PMC5852107

[67] Trumbower RD, Hayes HB, Mitchell GS, et al. Effects of acute intermittent hypoxia on hand use after spinal cord trauma: A preliminary study. Neurology 2017;89(18):1904–1907; doi: 10.1212/WNL.0000000000004596 28972191 PMC5664298

[68] Muter WM, Mansson L, Tuthill C, et al. A research protocol to study the priming effects of breathing low oxygen on enhancing training-related gains in walking function for persons with spinal cord injury: The BO2ST trial. Neurotrauma Rep 2023;4(1):736–750; doi: 10.1089/neur.2023.0036 38028272 PMC10659019

[69] McHugh LV, Miller AA, Leech KA, et al. Feasibility and utility of transcutaneous spinal cord stimulation combined with walking-based therapy for people with motor incomplete spinal cord injury. Spinal Cord Ser Cases 2020;6(1):104–109; doi: 10.1038/s41394-020-00359-1 PMC7688947 33239606

[70] Vivodtzev I, Tan AQ, Hermann M, et al. Mild to moderate sleep apnea is linked to hypoxia-induced motor recovery after spinal cord injury. Am J Respir Crit Care Med 2020;202(6):887–890; doi: 10.1164/rccm.202002-0245LE 32369393 PMC7491397

[71] Grossman RG, Fehlings MG, Frankowski RF, et al. A prospective, multicenter, Phase i matched-comparison group trial of safety, pharmacokinetics, and preliminary efficacy of riluzole in patients with traumatic spinal cord injury. J Neurotrauma 2014;31(3):239–255; doi: 10.1089/neu.2013.2969 23859435 PMC3904533

[72] Chow DSL, Nguyen A, Park J, et al. Riluzole in spinal cord injury study (RISCIS)-Pharmacokinetic (PK) sub-study: An analysis of pharmacokinetics, pharmacodynamics, and impact on axonal degradation of riluzole in patients with traumatic cervical spinal cord injury enrolled in the RISCIS Pha. J Neurotrauma 2023;40(17–18):1889–1906; doi: 10.1089/neu.2022.0499 37130044

[73] McKenna SL, Ehsanian R, Liu CY, et al. Ten-year safety of pluripotent stem cell transplantation in acute thoracic spinal cord injury. J Neurosurg Spine 2022;37(3):321–330; doi: 10.3171/2021.12.SPINE21622 35364569

[74] Fessler RG, Ehsanian R, Liu CY, et al. A phase 1/2a dose-escalation study of oligodendrocyte progenitor cells in individuals with subacute cervical spinal cord injury. J Neurosurg Spine 2022;37(6):812–820; doi: 10.3171/2022.5.SPINE22167 35901693

[75] Moritz C, Field-Fote EC, Tefertiller C, et al. Non-invasive spinal cord electrical stimulation for arm and hand function in chronic tetraplegia: A safety and efficacy trial. Nat Med 2024;30(5):1276–1283; doi: 10.1038/s41591-024-02940-9 38769431 PMC11108781

[76] NIH - National Center for Advancing Translational Sciences. NCATS Drug Repurposing: Seeking New Treatments Using Existing Drugs. 2023.

[77] Lieu B, Everaert DG, Ho C, et al. Skin and not dorsal root stimulation reduces hypertonus in thoracic motor complete spinal cord injury: A single case report. J Neurophysiol 2024;131(5):815–821; doi: 10.1152/jn.00436.2023 38505867

[78] Guertin PA. Preclinical evidence supporting the clinical development of central pattern generator-modulating therapies for chronic spinal cord-injured patients. Front Hum Neurosci 2014;8(MAY):272–217; doi: 10.3389/fnhum.2014.00272 24910602 PMC4038974

[79] Musselman KE, Shah M, Zariffa J. Rehabilitation technologies and interventions for individuals with spinal cord injury: Translational potential of current trends. J Neuroeng Rehabil 2018;15(1):40–48; doi: 10.1186/s12984-018-0386-7 29769082 PMC5956557

[80] Viele K, Berry S, Neuenschwander B, et al. Use of historical control data for assessing treatment effects in clinical trials. Pharm Stat 2014;13(1):41–54; doi: 10.1002/pst.1589 23913901 PMC3951812

[81] Kwon BK, Bloom O, Wanner IB, et al. Neurochemical biomarkers in spinal cord injury. Spinal Cord 2019;57(10):819–831; doi: 10.1038/s41393-019-0319-8 31273298

[82] Vose AK, Welch JF, Nair J, et al. Therapeutic acute intermittent hypoxia: A translational roadmap for spinal cord injury and neuromuscular disease. Exp Neurol 2022;347(September 2021):113891; doi: 10.1016/j.expneurol.2021.113891 34637802 PMC8820239

[83] Kirshblum S, Waring W. Updates for the international standards for neurological classification of Spinal Cord Injury. Phys Med Rehabil Clin N Am 2014;25(3):505–517, vii; doi: 10.1016/j.pmr.2014.04.001 25064785

[84] Katsoulakis E, Wang Q, Wu H, et al. Digital twins for health: A scoping review. NPJ Digit Med 2024;7(1):77–11; doi: 10.1038/s41746-024-01073-0 38519626 PMC10960047

[85] Atassi N, Berry J, Shui A, et al. The PRO-ACT database design, initial analyses, and predictive features. Neurology 2014;83(19):1719–1725; doi: 10.1212/WNL.0000000000000951 25298304 PMC4239834

[86] Mulcahey MJ, Jones LAT, Rockhold F, et al. Adaptive trial designs for spinal cord injury clinical trials directed to the central nervous system. Spinal Cord 2020;58(12):1235–1248; doi: 10.1038/s41393-020-00547-8 32939028

[87] Jones LAT, Bryden A, Wheeler TL, et al. Considerations and recommendations for selection and utilization of upper extremity clinical outcome assessments in human spinal cord injury trials. Spinal Cord 2018;56(5):414–425; doi: 10.1038/s41393-017-0015-5 29284795 PMC5951792

[88] Bolliger M, Blight AR, Field-Fote EC, et al. Lower extremity outcome measures: Considerations for clinical trials in spinal cord injury. Spinal Cord 2018;56(7):628–642; doi: 10.1038/s41393-018-0097-8 29700477 PMC6131138

[89] Catz A, Itzkovich M, Tesio L, et al. A multicenter international study on the spinal cord independence measure, version III: Rasch psychometric validation. Spinal Cord 2007;45(4):275–291; doi: 10.1038/sj.sc.3101960 16909143

[90] Sweatman WM, Heinemann AW, Furbish CL, et al. Modified PRISM and SCI-SET spasticity measures for persons with traumatic spinal cord injury: Results of a Rasch analyses. Arch Phys Med Rehabil 2020;101(9):1570–1579; doi: 10.1016/j.apmr.2020.05.012 32497601

[91] Tulsky DS, Kisala PA, Victorson D, et al. Overview of the spinal cord injury - quality of life (SCI-QOL) measurement system. J Spinal Cord Med 2015;38(3):257–269; doi: 10.1179/2045772315Y.0000000023 26010962 PMC4445018

[92] Velozo C, Moorhouse M, Ardolino E, et al. Validity of the neuromuscular recovery scale: A measurement model approach. Arch Phys Med Rehabil 2015;96(8):1385–1396; doi: 10.1016/j.apmr.2015.04.004 25912666

[93] University of Alabama at Birmingham. Spinal Cord Injury Facts and Figures at a Glance. Birmingham, AL; 2019.

